# Editorial: Bullying, cyberbullying, and dating violence: State of the art, evaluation instruments, and prevention and intervention proposals

**DOI:** 10.3389/fpsyg.2023.1119976

**Published:** 2023-03-30

**Authors:** Vanesa Martínez-Valderrey, Manuel Gil-Mediavilla, Mercedes Villasana-Terradillos, Sonia Alguacil-Sánchez

**Affiliations:** ^1^Faculty of Education of Palencia, University of Valladolid, Valladolid, Spain; ^2^Faculty of Humanities and Social Sciences, Comillas Pontifical University, Madrid, Spain

**Keywords:** bullying, cyberbullying, dating violence (DV), prevention, intervention, instruments, evaluation, prevalence

## Introduction

In recent years there has been an increase in research focused on the study of violent behavior, especially the one which occurs within educational and online environments (Hinduja and Patchin, 2015; Patchin and Hinduja, 2015; Wolke and Lereya, 2015). In this regard, the increase in aggressive behavior among peers or young couples generates alarming social concerns (Solberg and Olweus, 2003; Avilés and Monjas, 2005; Cerezo and Ato, 2005; Ramírez, 2006).

In this respect, works on peer bullying revolve around counseling programs, studies on victimization related to psychosocial maladjustment or mental health (Cole et al., 2016; Garaigordobil and Martínez-Valderrey, 2017; Salmerón and Inostroza, 2017), legal issues arising from these practices, controlled trials with school groups, correlation of these phenomena with suicide, etc. (Benítez et al., 2007; Perret et al., 2020; Buelga et al., 2022). Meanwhile, studies on dating violence make their way and are directed toward the definition of the construct and its prevention as well as the assessment of information and communication technologies (ICT) as mediators of this phenomenon.

Interestingly, the study of these behaviors is not recent but dates back to 1973 when Dan Olweus referred, for the first time, to the concept of violence between peers as *bullying*, and precisely from this moment on, the problem began to be given visibility (Olweus, 1993; Crick et al., 1999). Despite the 50 years that have passed since the publication of the first studies on this phenomenon, it seems that the efforts to prevent it either have not been enough or do not improve the numbers of those affected (Hinduja and Patchin, 2018). Likewise, the first approaches to dating violence were made by Makepeace (1981) in terms of physical assaults or threats that occur between two people in a dating relationship. In this timid approach to the phenomenon, only the physical violence exercised and the degree to which it affected the other partner were sought to be measured (Rodríguez-Caballero and Perdomo-Escobar, 2021).

Complementarily, the advent of information and communication technologies (ICTs) and the increase in their frequency of use and ease of access motivated the emergence of new forms of socialization, which are instantaneous and effective in their communication and relational aspects (Fox et al., 2014; Garaigordobil and Martínez-Valderrey, 2014a). However, with ICTs also came other forms of abuse such as cyberbullying, sexting, or online dating violence among other phenomena that proliferate as the expertise and imagination increase in those using technology for these purposes (Fox et al., 2014; Gámez-Guadix et al., 2018).

## Conceptualization and typologies

One of the challenges in conceptualizing phenomena related to violence is the lack of consensus among expert authors (Benítez and Justicia, 2006), the absence of clarity in determining violent behavior (Emery, 1989), or the individual bias to justify behavior (Rodríguez-Caballero and Perdomo-Escobar, 2021). In fact, defining an abusive act can depend upon judgment or social values (Jackson, 1999).

Nevertheless, one of the dimensions of peer abuse that the scientific community agrees on is that it is a specific conduct derived from aggressive behavior (Ascher, 1994; Espelage and Swearer, 2003). The following criteria are added to this dimension in order to consider the phenomenon (Olweus, 1993; Benítez and Justicia, 2006; Serrano, 2006): (1) there is a power imbalance between victim and perpetrator; (2) it is prolonged over time (repeated frequently); (3) there is an intention to harm the victim; (4) there is a varied nature of abuse (direct: physical; indirect: verbal, social, and psychological).

Cyberbullying, on the other hand, is defined as the use of ICTs to intentionally and repeatedly cause harm to another person (Smith et al., 2008; Peter and Petermann, 2018). The specific characteristics of this type of abuse include (Almeida et al., 2008; Dehue et al., 2008; Huang and Chou, 2010; Pornari and Wood, 2010): (1) a lack of real awareness of the harm caused; (2) crossing of spacetime boundaries to exercise and experience harassment; (3) an increase in the observing public; and (4) the disinhibition effect (Livingstone, 2008; Suler, 2008). The types of cyberbullying that are most frequently expressed in scientific works of literature are (Kowalski et al., 2010): (1) electronic insults, (2) harassment (repeatedly sending offensive messages in different virtual spaces), (3) denigration (digitally sharing derogatory and false information about a person), (4) identity theft, (5) disclosure (revealing private information about a person), (6) exclusion, (7) cyberstalking, and (8) happy slapping (recording a physical attack and uploading it to the internet).

Violence in dating is defined as aggressive behaviors aimed at controlling and dominating the partner (Wekerle and Wolfe, 1998; Celis-Sauce and Rojas-Solís, 2015; Martínez et al., 2016). Within the types of violence in dating, the following are distinguished (Rey-Anacona, 2013; Rodríguez-Caballero and Perdomo-Escobar, 2021): (1) physical, (2) emotional or psychological, (3) sexual, (4) economic, and (5) cyber violence. In turn, authors like Darvell et al. (2011) suggest the following subtypes within violence exercised through electronic means: (1) hostility (posting or sending threatening messages *via* social networks, text messages, or email), (2) intrusiveness (monitoring received messages, changing passwords, or creating fake profiles), (3) humiliations (text or graphic publications), and (4) exclusion (deletion or blocking on social networks or friend lists).

In light of the definitions provided about each of the phenomena in its different adopted forms, it can be stated that one of the common links is the explicit objective of intentionally causing pain and suffering to a victim and controlling, in some way, their behavior.

## Models and explanatory theories of violent behavior

The literature review highlights the difficulty detected along the process of instrumentalizing or operationalizing the definitions of phenomena for determining a holistic explanatory model of violence from any of the expressions analyzed so far (Kowalski et al., 2014; Estévez et al., 2019; Ansary, 2020).

It is because, traditionally, violent behavior has been attempted to be explained generally from three perspectives: (1) biological (biological foundation: genetic and environment), (2) psychological (way of interpreting and evaluating received stimuli: values, beliefs, and emotional state), and (3) sociological (sociocultural factors).

However, in addition to the classical theories, some of the explanatory models of violent behavior that are currently being reviewed are (Ansary, 2020):

1) The BGCM-Barlett and Gentile Cyberbullying Model (Barlett et al., 2021). It states that anonymity in cyberspace and lack of power imbalance (from a physical perspective) lead to positive attitudes toward bullying and are predictors of cyberbullying perpetration.
2) The I3 (I-cubed) model (Slotter and Finkel, 2011). It identified factors that predict intimate partner violence, such as (1) triggering factors, (2) promoter factors, and (3) factors that reduce aggression. The model was also adapted to measure cyberbullying perpetration behaviors (Wong et al., 2018). Despite its explanatory power, the study participants were a reduced sample (university students in Hong Kong), and the definition was too broad.
3) The Swearer and Hymel (2015) social-ecological diathesis-stress model. It recognizes the dynamic interaction between genetic, social, and environmental factors to explain violent behaviors.

In the same way, currently, other models combining elements from different theories may contribute to establishing a supporting model for violence. The Mental Sponge theory is one of these emerging models that has been shown to be effective in several psychosocial studies (Vuong and Napier, 2015). This theory focuses on explaining how a person incorporates new values into his or her way of thinking if they are compatible with the existing information in his or her mentality (Vuong et al., 2022). Based on the evidence in neuroscience, the model considers that the creation of the thought is based on the processing of information: from basic biochemical reactions to genetic influence and from instinct to advanced cognition (Vuong, 2023).

## Objectives

The interest in studying bullying, cyberbullying, and dating violence has increased due to the consequences faced by those who suffer from it, with suicide or death being the harshest outcome. As seen in current works of literature, it is necessary to unify criteria regarding the conceptualization of bullying and cyberbullying (Ansary, 2020), to deepen the definition of violence in dating (Gámez-Guadix et al., 2018), and to design assessment instruments with adequate psychometric guarantees of reliability and validity (Selkie et al., 2015; Salmerón and Inostroza, 2017) as well as to create intervention programs to prevent their occurrence (Garaigordobil and Martínez-Valderrey, 2014b, 2018).

The objective of this Research Topic is to show the current state of the problem of bullying and dating violence whether in-person or online and to contribute to the existing literature on these phenomena.

## Results and conclusions

In this Research Topic, there are five studies that deal with the relationships established between bullying, cyberbullying, and dating violence with different predicting and mediating variables, as well as the results of an intervention program to promote the establishment of healthy intimate relationships.

The results of the study “*Longitudinal relationship between bullying victimization and depression among left-behind children: roles of negative thoughts and self-compassion*” (Yan et al.) showed that victimization through bullying acted as a predictor of childhood depression, negative thoughts and self-pity being the mediating variables in the relationship established between bullying and depression. In the same way, Eroglu et al. in their research “*Cyber victimization and well-being in adolescents: The sequential mediation role of forgiveness and coping with cyberbullying*” found negative correlations between cyber victimization and forgiveness and between coping with these behaviors and wellbeing. Additionally, in this study, forgiveness and coping strategies for cyberbullying were mediating variables in the relationship established between cyber victimization and wellbeing. On the other hand, Zhao and Yu in “*A meta-analytic review of moral disengagement and cyberbullying*” supported the hypothesis of the influence of moral disconnection on the perpetration of cyberbullying behaviors, in this case, age, gender, and cultural background being the mediating variables.

Regarding dating violence, the results of the study “*Evaluating female experiences of electronic dating violence in jordan: motivations*” (Alsawalqa), developed with a sample of 104 Arab women, contribute to consider the hypothesis that considers that the victims of partner violence exerted through digital technologies actually increased the probability of suffering psychological, emotional, verbal, and physical violence. In this same order of things, prevention is presented as an effective strategy to reduce violent behavior in any of its forms. An example to prevent and mitigate dating violence is “*PRO-Mueve Relaciones Sanas – A gender-based violence prevention program for adolescents: assessment of its efficacy in the first year of intervention*” (Velasco et al.). This program, in its first year of application, has proven to significantly reduce both behaviors of benevolent sexism and myths derived from romantic love, whereas it increased the knowledge of gender violence.

The results of the studies presented in this Research Topic show that the consequences of face-to-face and online bullying among peers and dating violence are disastrous for those who suffer from it, and at the same time show a close relationship with mental health. In this regard, it should be noted that the development of balanced social and emotional behavior as well as the establishment of healthy interpersonal relationships have a determining weight on the correct psychological, cognitive, and affective development of schoolchildren and, by extension, of future societies.

The school as a reference context in the establishment of interpersonal relationships between peers has a crucial role in the implementation of interventions that are committed to developing values and creating a healthy coexistence framework that reduces the risks associated with violent behavior in any of its manifestations.

## Author contributions

All authors listed have made a substantial, direct, and intellectual contribution to the work and approved it for publication.
